# A randomized controlled trial to assess the efficacy of an interactive mobile messaging intervention for underserved smokers: Project ACTION

**DOI:** 10.1186/1471-2458-12-696

**Published:** 2012-08-25

**Authors:** Damon J Vidrine, Faith E Fletcher, Heather E Danysh, Salma Marani, Jennifer Irvin Vidrine, Scott B Cantor, Alexander V Prokhorov

**Affiliations:** 1 Department of Behavioral Science, The University of Texas MD Anderson Cancer Center, P.O. Box 301439, Unit 1330, Houston, TX, 77030-1439, USA; 2 Department of Health Disparities Research, The University of Texas MD Anderson Cancer Center, P.O. Box 303906, Unit 1440, Houston, TX, 77030-3906, USA; 3 Department of Biostatistics, The University of Texas MD Anderson Cancer Center, P.O. Box 301402, Unit 1411, Houston, TX, 77230-1402, USA

**Keywords:** Smoking cessation, mHealth, Underserved populations, Community outreach, Mobile clinic

## Abstract

**Background:**

Despite a significant decrease in smoking prevalence over the past ten years, cigarette smoking still represents the leading cause of preventable morbidity and mortality in the United States. Moreover, smoking prevalence is significantly higher among those with low levels of education and those living at, or below, the poverty level. These groups tend to be confronted with significant barriers to utilizing more traditional smoking cessation intervention approaches. The purpose of the study, Project ACTION (Adult smoking Cessation Treatment through Innovative Outreach to Neighborhoods), is to utilize a mobile clinic model, a network of community sites (i.e., community centers and churches) and an interactive mobile messaging system to reach and deliver smoking cessation treatment to underserved, low-income communities.

**Methods/Design:**

We are using a group-randomized design, with the community site as the sampling unit, to compare the efficacy of three smoking cessation interventions: 1) Standard Care - brief advice to quit smoking, nicotine replacement therapy (NRT), and self-help materials; 2) Enhanced Care - standard care components plus a cell phone-delivered text/graphical messaging component; and 3) Intensive Care - enhanced care components plus a series of 11 cell phone-delivered proactive counseling sessions. An economic evaluation will also be performed to evaluate the relative cost effectiveness of the three treatment approaches. We will recruit 756 participants (252 participants in each of the 3 intervention groups). At the time of randomization, participants complete a baseline assessment, consisting of smoking history, socio-demographic, and psychosocial variables. Monthly cell phone assessments are conducted for 6 months-post enrollment, and a final 12-month follow-up is conducted at the original neighborhood site of enrollment. We will perform mixed-model logistic regression to compare the efficacy of the three smoking cessation intervention treatment groups.

**Discussion:**

It is hypothesized that the intensive care approach will most successfully address the needs of the target population and result in the highest smoking cessation rates. In addition to increasing cessation rates, the intervention offers several features (including neighborhood outreach and use of mHealth technology) that are likely to reduce treatment barriers while enhancing participant engagement and retention to treatment.

**Trial registration:**

This randomized controlled trial is registered with clinicaltrials.gov registration number NCT00948129.

## Background

Cigarette smoking remains the largest preventable cause of death in the United States (US), accounting for an estimated 443,000 premature deaths per year [1]. It is well-documented that cardiovascular disease (29%), respiratory disease (23%), lung cancer (28%), cancers other than lung (11%), secondhand smoke exposure (11%), and other diseases (<1%) account for all smoking-related deaths [1]. The US is a global leader in tobacco control efforts, and trends indicate that smoking prevalence has decreased over the past several decades. However, pronounced health disparities still exist in access to tobacco control resources and smoking cessation programs, resulting in fewer economically disadvantaged individuals successfully quitting smoking as compared with middle- and high-income individuals [2]. Given the adverse health outcomes associated with smoking and data indicating that fewer economically disadvantaged individuals access and utilize smoking cessation services, identifying effective practices to address tobacco-related disparities are critical.

In addition to unequal access to smoking cessation services, it is well-established that the tobacco industry aggressively markets its products to members of racial/ethnic minority groups, economically disadvantaged individuals, and urban residents [3]. These groups tend to be confronted with significant barriers to utilizing traditional smoking cessation intervention approaches. Despite striking tobacco-related disparities among disadvantaged populations, few studies targeting low-income smokers have incorporated racial/ethnic minorities into the sample [4-7]. Additionally, few interventions exist to increase the range of smoking cessation services to individuals who lack access to transportation, have limited or no medical insurance, and typically do not utilize traditional health care facilities. Hence, employing innovative strategies to increase accessibility, availability, and affordability of smoking cessation services particularly among disadvantaged populations is a national public health priority [8,9].

The overarching goal of this National Cancer Institute R01-funded study (R01CA141628) is to deliver and evaluate a novel smoking cessation program targeted to low-income uninsured and underinsured individuals. “Delivering” in the context of this proposal means taking the program directly to neighborhoods where the target audience dwells. By utilizing a mobile clinic model and a network of community sites, we increase the range of cancer prevention services for individuals with limited transportation abilities and limited or no medical insurance and are therefore unlikely to use traditional health care facilities. The specific aims of the study are to: 1) compare the efficacy of three smoking cessation interventions targeting community based low-income uninsured and underinsured individuals in a group-randomized trial; 2) evaluate the role of quit motivation, nicotine withdrawal, risk perception, self-efficacy, social support, and negative affect as potential mediators of smoking abstinence; and 3) evaluate the cost-effectiveness of the three treatment conditions.

### Addressing smoking-related disparities through innovative approaches

Income and education level are among the strongest predictors of both smoking prevalence and successful cessation [10]. Comparisons of smoking prevalence data over 25 years reveal a widening gap between those at the lowest and highest income levels [11]. Recent national estimates indicate that the prevalence of smoking is highest among those individuals with less than a high school education (28.4%), those with no health insurance (28.6%), those living below the federal poverty level (27.7%), and those between the ages of 18–24 (23.8%) [12]. Furthermore, research suggests that compared to higher-SES smokers, low-income smokers have limited resources for quitting smoking and are less likely to receive assistance through the health care system [2]. Therefore, it is critically important to expand the reach of evidenced-based smoking cessation and treatment resources to population subsets with low levels of education and income.

Mobile health clinics offer a promising model for providing care to underserved populations with limited access to traditional health care services. These clinics have provided the following outreach services: acute and chronic care treatment, immunizations, dental exams, post-disaster care, prenatal care, screenings for mental health, cervical and breast cancer screenings, hearing tests, and nutrition counseling [13-21]. Mobile clinics are gaining popularity, but there are few data on outcomes beyond utilization rates. Results of a case–control study examining outcomes for prenatal care among uninsured immigrants indicated that patients using the mobile clinic received prenatal care significantly earlier than participants using prenatal care from a stationary clinic. Prenatal care on the mobile clinic was compared to stationary clinics and similar positive outcomes were found (e.g., gestational age at delivery, birth weight) [22]. Post-test knowledge and skills for 777 participants were significantly increased after a breast cancer screening and awareness effort delivered by a mobile clinic [23]. Although we identified no literature on using mobile clinics to provide smoking cessation services, the evidence from other health conditions suggests that a mobile clinic approach has great potential. A clear need exists to test the efficacy, utilization, and cost-effectiveness of using a mobile clinic to promote access to smoking cessation services among low-income smokers.

As cell phone use has become more widespread [24], efforts to use mobile technology for health-related interventions (mHealth) have greatly increased. For many economically disadvantaged individuals, a cell phone may be the only technological device with basic computing capabilities. For example, preliminary results from the Centers for Disease Control and Prevention (CDC) National Health Interview Survey (NHIS) indicate that adults living in poverty (30.9%) or near poverty (23.8%) were more likely to be living in households with only wireless telephones than higher income adults (16.0%) [25]. Wireless-only adults were also more likely to be current smokers (30.6%) than were adults living in landline households (18.0%); wireless-only users were additionally more likely to be uninsured (28.7%) compared to those residing in landline households (13.7%). Finally, compared with adults living in households with landlines, wireless-only adults were more likely to have experienced financial barriers to obtaining needed health care, and they were less likely to have access to routine medical care [25].

Several recent smoking cessation studies utilizing cell phones and text messaging have shown feasible and promising results [26-30]. Studies in other areas including alcohol prevention, diabetes education, sexual health, diet and physical activity have also shown promising results [31-35]. Findings from the Pew Research survey indicate that 17% of cell phone owners have used their phones to find medical information, and 9% have downloaded an application to help track or manage their health [24]. Hence, widespread cell phone use offers public health experts the opportunity to disseminate programs and interventions to populations by capitalizing on their extant adaptation and utilization of mobile technology as a vehicle to improve health outcomes.

## Methods/Design

### Smoking cessation intervention approaches

Project ACTION (Adult smoking Cessation Treatment through Innovative Outreach to Neighborhoods) is currently underway. In this study, we are employing a group-randomized design, with the neighborhood site as the sampling unit, to compare the efficacy of three smoking cessation interventions: 1) Standard Care (SC) - brief advice to quit smoking, nicotine replacement therapy (NRT), and self-help materials; 2) Enhanced Care (EC) - standard care components plus a cell phone-delivered text/graphical messaging component; and 3) Intensive Care (IC) - enhanced care components plus a series of 11 cell phone-delivered proactive counseling sessions. We have taken an expanded approach in the design of these interventions, which allows us to assess the benefit of adding novel components to a well-recognized standard care treatment approach. An important contribution of the current study is the assessment of these interventions in the context of a mobile smoking cessation clinic, which targets underserved smokers in their own communities.

### Participants

The University of Texas MD Anderson Cancer Center Institutional Review Board approved the study. To be eligible for the study, participants must be 18 years of age or older; have smoked at least 100 cigarettes; be English or Spanish speaking; smoke at least 5 cigarettes per day, on average; and be willing to set a quit smoking date within a week from the date of enrollment. Exclusion criteria are as follows: a positive history of a medical condition that precludes use of the nicotine patch; current use of NRT or other smoking cessation medications (e.g., Chantix or Zyban); current enrollment in another smoking cessation program; and currently pregnant or breastfeeding.

### Procedures

#### Recruitment

Participants are recruited from various neighborhood sites (i.e., community centers and churches) located throughout the large metropolitan area of Houston, Texas. During participant recruitment at each of the neighborhood sites, the research staff is available to explain the smoking cessation study to potential participants and to answer questions. The research staff then individually obtains informed consent from those who are eligible and interested in enrolling in the smoking cessation trial. At the time of enrollment, participants complete a baseline assessment, consisting of smoking history, socio-demographic, psychosocial, and health literacy questions. Monthly cell phone assessments are conducted for 6 months post-study enrollment, and a final 12-month follow-up assessment is conducted in-person at the original neighborhood site of enrollment.

#### Baseline assessment

Research staff administers an audio computer assisted self-interview (ACASI) to all participants. The interview takes approximately 30–45 minutes to complete and answers are recorded directly into a computerized database that contains programmed logic checks and skip patterns. After completing the entire baseline assessment, participants are compensated with a $20 gift card.

#### Cell phone assessments

Assessments will be administered monthly for the first 6 months post-study enrollment. These brief assessments collect information on the participants’ attitudes about smoking (i.e., risk perceptions/outcome expectancies and self-efficacy for quitting), daily coping, nicotine withdrawal symptoms, nicotine patch use, and current smoking status. To ensure the feasibility of this methodological approach, we provide cell phones to all participants. The cell phones are part of a phone plan that includes voice capabilities to allow for completion of the brief assessments over the cell phone. Participants who complete these assessments are compensated with an additional $10 gift card for each completed monthly cell phone assessment. Following completion of the study, participants are allowed to keep the phones.

#### 12-month follow-up

The primary outcome is smoking status at the 12-month follow-up. Research staff return to each community site approximately 12-months after the initial visit to administer the 12-month follow-up assessment. The 12-month assessment mirrors the baseline assessment. Smoking status is biochemically confirmed using saliva cotinine and expired carbon monoxide. Participants receive a $40 gift card after completing this assessment.

##### Conceptual framework

**Figure 1:**
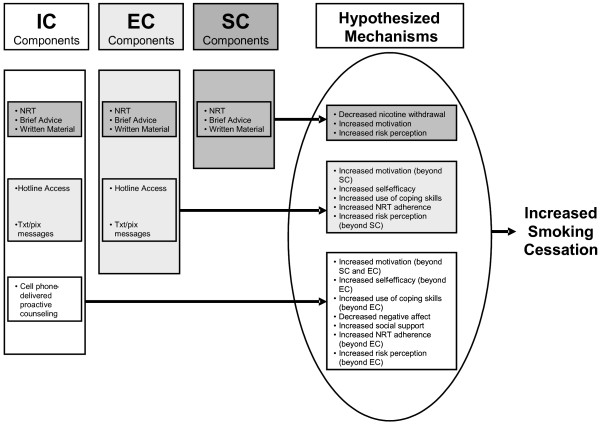
Conceptual framework for hypothesized treatment mechanisms.

A conceptual framework illustrating hypothesized mechanisms through which the interventions increase smoking cessation is presented in Figure 1. This framework compares and contrasts the treatment components of the three groups (SC, EC, and IC) and depicts the manner in which each treatment component exerts an effect on cessation. The individual treatment components are further described below.

### Intervention components

#### Nicotine replacement therapy (NRT)

NRT in the form of nicotine patches is provided with detailed instructions on proper use to reduce nicotine withdrawal symptoms and enhance cessation outcomes. The provision of NRT is considered part of the minimum recommended level of acceptable care [36]. Studies have demonstrated that the use of the nicotine patch approximately doubles abstinence rates compared to placebo [37]. Although nicotine patches are available over-the-counter, we provide them in order to ensure proper monitoring of patch use and to reduce potential economic barriers. All eligible participants are provided with a 10-week supply of nicotine patches at no cost, including 21 mg patches for 6-week use, 14 mg patches for 2-week use, and 7 mg patches for 2-week use.

#### Brief advice to quit smoking

Trained research staff members provide brief advice to quit smoking to all participants. Brief advice to quit smoking is regarded as an effective and cost-efficient method to reduce smoking [38]. Even when the advice is limited to 3 minutes or less, a significant impact is seen [36]. As recommended by the Public Health Service Guideline, brief advice to quit smoking involves the provision of a clear, strong, and personalized message to quit and offer assistance in setting a quit date [36].

#### Self-help written materials

All participants are provided written self-help materials. The materials advise participants to quit smoking, list adverse health effects of smoking, and recommend the use of social support and coping skills. Meta-analyses indicate that self-help materials, when provided with other forms of cessation treatment, increase smoking abstinence rates [36].

#### Access to a hotline

Individuals in the EC and IC groups are provided a hotline number where they can reach the study counselors. The hotline number is programmed into each cell phone to facilitate easy use. Although past hotline studies have had limited success [39,40], the current hotline has some enhancements that are designed to facilitate the use of the hotline. Specifically, the hotline is staffed by counselors who are familiar with the study, including the recruitment process and the text messaging component. In addition, the use of cell phones allows access to hotline support at the time of urge or following a lapse. The hotline is managed by project counselors who record participants’ use of the hotline, including the number of times each participant calls, reason for calls, length of calls, and topic areas discussed in calls.

#### Cell phone delivered text messages and picture messages

Introducing cessation treatment within the neighborhood sites through a mobile clinic is an important step. However, the intensity of the treatment that can feasibly be provided in a single mobile clinic visit is limited. Additionally, time constraints associated with the neighborhood site visit will also limit the provision of support, teaching of coping skills, and general relapse prevention assistance. The literature indicates that most individuals who quit smoking relapse within the first week of abstinence [41], and that increasing treatment intensity decreases relapse rates [36]. Therefore, when time limitations are combined with the infrequency of site visits, the need for an extension of treatment outside of a single mobile clinic visit is clear. We address these issues by providing an interactive text and picture messaging intervention to participants in the EC and IC groups.

Participants randomized to the interactive text messaging groups (EC & IC) receive a series of text messages scheduled to begin shortly prior to their scheduled quit date, and continuing for a 3-month period. In the first week of treatment, participants receive 5 messages per day. The frequency tapers off to one message per day by week 4, and continues at this frequency until week 12. The text and picture messages are received by the participant on the cell phone provided to them by the project research staff.

The messages are designed to increase cigarette smoking and health knowledge, maintain/increase quit motivation, promote coping skills use, increase NRT compliance, increase social support, and enhance self-efficacy to quit smoking. The content of the messages is designed to fit into one of four different categories: 1) problem solving/coping skills; 2) knowledge/risk perception; 3) increasing and maintaining quit motivation; and 4) increasing social support. Additionally, to address the specific needs of each participant, the text messages are tailored on four levels: 1) smoking status; 2) disease history; 3) concern of future disease; and 4) preferred coping skills. These characteristics are ascertained through baseline data collection. Text messaging content is primarily drawn from cognitive-behavioral and Motivational Interviewing (MI) techniques.

#### Cell phone delivered proactive counseling

The only distinction between the IC and EC treatment approach is proactive counseling. Participants in the IC group receive 11 proactive counseling sessions over a 12-week period. Substantial evidence indicates a strong dose–response relationship between intensity of treatment and cessation success, with the first week after quitting being a critical period [41-43]. This relationship is hypothesized to be driven primarily by the concentrated and sustained social support provided to individuals. As with text messaging, counseling session content is primarily drawn from cognitive-behavioral and MI techniques. Session topics include coping with withdrawal, maintaining a commitment to continued abstinence, and relapse prevention (see Table 1). Each participant is assigned to a counselor (who will make the majority of calls to that participant) in order to optimize rapport and familiarity with participants’ unique concerns and smoking history. The number of calls completed, length of calls, and topic areas covered in each call is recorded by the counselor.

**Table 1:** Schedule of calls and call content

**Call**	**Time of Call**	**Content of Call**
1	1 day prior to quit date	Preparing to quit - why quit and making the commitment to quit
2	On quit date	Quitting Smoking – getting through the first day
3	2 days post quit date	Surviving Withdrawal - withdrawal facts and coping skills
4	4 days post quit date	Managing high risk situations
5	7 days post quit date	Stress, Negative Affect & Smoking
6	10 days post quit date	Improving Support and Asserting Yourself
7	2 weeks post quit date	Reviewing Problem Solving & Dealing with Lapses
8	4 weeks post quit date	Reinforcing Benefits of Being a Nonsmoker
9	6 weeks post quit date	Maintaining Commitment – Keeping Motivated
10	9 weeks post quit date	Successes and Challenges in Smoking Cessation
11	12 weeks post quit date	Long-Term Relapse Prevention

### Study measures

#### Quit motivation

Quit motivation is assessed with the Reasons for Quitting questionnaire. This 20-item scale assesses intrinsic (health concerns, self-control) and extrinsic (immediate reinforcement, social influence) motives for quitting smoking. Both intrinsic motives and the ratio of intrinsic to extrinsic motives have been demonstrated to predict successful smoking cessation [44,45].

#### Risk perception

To assess risk perceptions and outcome expectancies, we use items based on recommendations by Brewer and colleagues [46]. Specifically, participants respond to a series of four questions: 1) “If you don’t quit or go back to smoking, what are your chances of ever developing a smoking-related health problem?”; 2) “If you quit smoking or remain quit, what are your chances of ever developing a smoking-related health problem?”; 3) Compared to other smokers, what are your chances of ever developing a smoking-related health problem if you continue or go back to smoking?”; 4) Compared to other smokers, what are your chances of ever developing a smoking-related health problem if you quit smoking or remain quit?”

#### Self-efficacy

A 9-item scale developed and validated by Velicer and colleagues is administered to assess self-efficacy [47]. This commonly used self-efficacy scale assesses an individual’s confidence in his/her ability to avoid smoking in a variety of situations.

#### Intention to quit smoking

Intention to quit smoking is assessed by asking participants whether or not they plan to quit smoking. Responses are entered on a 7-point scale, ranging from 1, definitely no, to 7, definitely yes. An additional intention item considers time frame (i.e., quitting within the next month or within the next year). Similar items are used to assess intention to cut-back on the number of cigarettes smoked per day, and the time frame for planned reduction.

#### Contemplation Ladder

The Contemplation Ladder is a single item that asks respondents to circle a number on a 10-rung ladder that represents their current level of readiness to consider quitting smoking. Responses range from 0 (no thought of quitting) to 10 (taking action to quit smoking, e.g., cutting down enrolling in a cessation program) [48].

#### Smoking status

A 10-item questionnaire is used to assess smoking behavior, including point prevalence abstinence within the last 24 hours, 7 days, 30 days, and since the time of last contact. Cigarettes smoked per day, longest period of abstinence since last contact, number of relapses, use of nicotine replacement, exposure to other types of tobacco, or use of any other cessation treatment (e.g., professional assistance and self-help) are also included [49].

#### Demographic, health, and smoking questionnaires

These items are designed to collect information on demographic characteristics (e.g., age, race/ethnicity, education level, income, and occupation), current medications, current medical care (including number and type of healthcare visits), drug/alcohol use, history of depression, and smoking history (e.g., years smoked, amount smoked, age of initiation, previous quit attempts, and relapse history). These items have been used in several of our other smoking cessation trials as well as in our pilot study [50-52].

#### Fagerström Test for Nicotine Dependence (FTND)

The original items of the Fagerström Tolerance Questionnaire (FTQ) were derived from theoretical conceptualizations of reliance on nicotine [53]. The instrument is reliable and useful in a broad spectrum of populations [54]. The *FTND*, a modification of the FTQ, is a 6-item scale with solid psychometric properties [55].

#### Center of Epidemiologic Studies Depression Scale (CES-D)

The CES-D is a 20-item measure developed to assess depressive symptoms in community non-clinical populations [56]. This scale consists of four major factors: depressed affect, enervation, lack of positive affect, and interpersonal problems. Good psychometric properties have been demonstrated across diverse populations [57].

#### Positive and Negative Affect Schedule (PANAS)

The PANAS is a 20-item adjective rating form that includes both positive and negative affect scales. Ratings are based on a 5-point Likert scale (1 = very slightly or not at all to 5 = extremely). The PANAS has demonstrated excellent reliability and validity properties [58].

#### Interpersonal Support Evaluation List (ISEL)

The ISEL is used to measure social support. This 12-item measure assesses three constructs of social support: tangible, appraisal, and belonging [59]. Social support is a well-established predictor of successful smoking cessation [36].

#### Wisconsin Smoking Withdrawal Scale (WSWS)

The WSWS is used to measure smoking withdrawal symptoms [60]. It includes subscales for anger, anxiety, sadness, concentration difficulty, craving, hunger, and sleep. All subscales have excellent internal consistency and demonstrate clear increases in withdrawal [61].

#### Daily Coping Inventory

This is a 9-item scale that measures positive and negative aspects of cognitive and behavioral coping skills [62].

#### American Thoracic Society’s Respiratory Symptoms Assessment

Respiratory symptoms are characterized by the occurrence of specific symptoms (morning and daytime cough, phlegm production, wheezing, chest tightness and pain) scored on the standard American Thoracic Society’s respiratory symptom assessment, an 8-item measurement tool. For each participant, the overall respiratory symptom score and individual symptom scores will be compared with normative scores computed from nonsmokers [63].

#### Wisconsin Inventory of Smoking Dependence Motives (WISDM)

The WISDM is a 37-item scale that measures smoking motivations and nicotine dependence across 11 subscales [64,65].

#### Minnesota Nicotine Withdrawal Scale (MNWS)

The MNWS is a 15-item scale that measures smoking withdrawal symptoms including anger, anxiety, cravings, depression, difficulty concentrating, hunger, impatience, insomnia, and restlessness [66].

#### Subjectively Measured Second-Hand Smoke Index

A subjective measure of reduction of in-home smoking is assessed by self-report based on the Home Environmental Tobacco Smoke Index [67]. The Home Environmental Tobacco Smoke Index has 9 items and asks informants if smoking is permitted in their home and how many people typically smoke in their home.

#### Subjective Social Status (SSS) Ladders (SES and Community)

Each of the two ladders comprises 1 item. The SES SSS ladder assesses how an individual perceives his/her SES compared to the general U.S. population; the Community SSS ladder assesses how the participant perceives his/her SES compared to his/her community [68].

#### Perceived General Health

The 1-item measure from the CDC’s Behavioral Risk Factor Surveillance System (BRFSS) is used to assess the participant’s perception of his/her general overall health status [69].

#### Perceived Stress Scale (PSS)

The 4-item PSS is used to assess the degree to which respondents find their lives to be stressful [70].

#### Assessment of Health Literacy

Participant health literacy is assessed using the short version of the Test of Functional Health Literacy in Adults (S-TOFHLA) [71] and the Rapid Estimate of Adult Literacy in Medicine (REALM) [72] (or for Spanish speakers, the Short Assessment of Health Literacy for Spanish Adults (SAHLSA) [73], which is based on the English REALM). Like many other health literacy instruments, these assessments must be administered in-person by research staff, and therefore will not be part of the assessment that is delivered using the ACASI system described above. Health literacy will be assessed at the baseline visit.

### Data analysis

The focus of Aim 1 is to compare the efficacy of three smoking cessation interventions (SC, EC, and IC), in which a generalized linear mixed-modeling approach will be used with logistic regression. Seven-day abstinence at the 12-month follow up will be evaluated as the primary outcome. In these analyses, each neighborhood site will be modeled as a random effect nested within each treatment condition to account for clustering, and the treatment condition will be modeled as a fixed effect. The regression coefficient associated with the treatment group variable in this analysis will represent the overall log odds of being abstinent in the EC group relative to the SC group, or the IC group relative to the EC group at the 12-month follow-up, controlling for covariates. An intent-to-treat approach will be used in which participants who do not complete the 12-month follow-up assessment will be coded as smokers.

Aim 2 is to evaluate the role of quit motivation, nicotine withdrawal, risk perception, self-efficacy, social support, and negative affect as potential mediators of smoking abstinence. Structural equation modeling (SEM) will be used to develop a model representing the patterns of association among the mediating variables, intervention method and abstinence. SEM provides a flexible approach to modeling means, covariance, and correlation structures that yields relevant effect estimates (directional and non-directional; direct, indirect, and total) and standard errors for all of the parameters of interest in mediator analyses.

Aim 3 is to evaluate the cost-effectiveness of the three treatment conditions. We will perform an incremental cost-effectiveness analysis by comparing the expected economic costs and clinical benefits of the three strategies. We will create a decision-analytic model using TreeAge Pro 2012 software to conduct the incremental cost-effectiveness analysis. Although we will only have quit rates after 12 months of the study interventions, we will use the model to predict the lifetime costs (measured in dollars) and benefits (measured in quality-adjusted life expectancy) of the three smoking cessation strategies. We will then use sensitivity analysis to determine if the conclusions of the analysis are robust based on variation in the model’s parameters.

### Power considerations

The study uses a group-randomized design with repeated observations at baseline, monthly for the first 6 months post intervention, and at 12 months post intervention. A total of 126 neighborhood sites will be stratified according to type (community center vs. church) and ethnic composition, and randomly assigned from within strata to one of three treatment conditions: SC, EC, or IC conditions. This will yield 42 centers per condition. Sample size was estimated using parameters obtained from the published literature and from our own research [1,74]. The total sample size will be 756 (252 participants in each of the 3 treatment conditions) which allows for 95% power to detect a net difference of 11% or larger between the treatment groups and accounts for multiple comparisons for the three pairwise comparisons.

## Discussion

**Figure 2:**
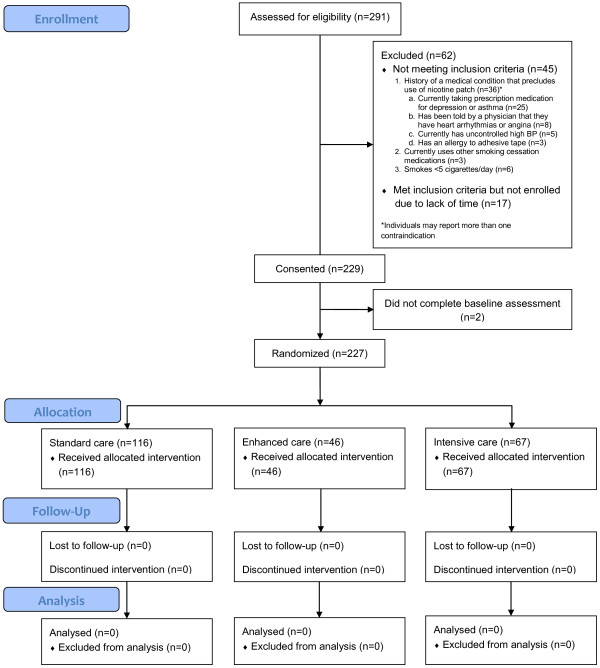
CONSORT diagram: Project ACTION.

Few controlled, randomized long-term smoking cessation interventions exist to reach groups that tend to be confronted with significant barriers to utilizing more traditional smoking cessation intervention approaches. Project ACTION delivers smoking cessation treatment to underserved individuals by utilizing cutting-edge mobile phone technology (voice, text messaging and multimedia graphical illustrations) to boost treatment intensity while limiting participant burden. Although Project ACTION is a novel approach conducted with an underrepresented and understudied population, our current recruitment and retention rates are promising (n = 227). See Figure 2 for the CONSORT (Consolidated Standards of Reporting Trials) diagram which displays participant study enrollment to date. Thus far, we have successfully recruited from 14 neighborhood sites and have enrolled a subset of individuals who are at greater risk of smoking maintenance given their limited access to smoking cessation services. Study participants are predominantly African American/Black (78.4%), have completed approximately 11.4 years of formal education, and half of the sample is unemployed (52.4%) and has an annual household income of $10,000 or less (46.7%). See Table 2 for complete demographic characteristics of the current sample. By employing a mobile health clinic model, tapping into existing community networks, and utilizing an interactive mobile messaging system, we have attempted to minimize the barriers that typically hinder individuals from both accessing health care and participating in research studies.

**Table 2:** Demographic characteristics of sample

**Characteristic**	**Standard Care (n=114)**		**Enhanced Care (n=46)**		**Intensive Care (n=67)**		**Total (n=227)**	
Mean age in years (SD)	46.9	(12.7)	48.3	(12.7)	47.4	(11.7)	47.3	(12.3)
Male, n (%)	53	(46.5)	23	(50.0)	26	(38.8)	102	(44.9)
Married/living with significant other, n (%)	28	(24.6)	22	(47.8)	20	(29.9)	70	(30.8)
Race/ethnicity, n (%)								
White	7	(6.1)	4	(8.7)	9	(13.4)	20	(8.8)
African American/Black	96	(84.2)	33	(71.7)	49	(73.1)	178	(78.4)
Hispanic/Latino	8	(7.0)	5	(10.9)	7	(10.5)	20	(8.8)
Other	3	(2.6)	4	(8.7)	2	(3.0)	9	(4.0)
Mean years of formal education (SD)	11.3	(3.0)	11.9	(2.8)	11.4	(3.3)	11.4	(3.1)
Education level, n (%)								
Less than high school	30	(26.3)	11	(23.9)	13	(19.4)	54	(23.8)
High school or equivalent	52	(45.6)	20	(43.5)	29	(43.3)	101	(44.5)
More than high school	32	(28.1)	15	(32.6)	25	(37.3)	72	(31.7)
Current work status, n (%)								
Working full or part time	56	(49.1)	21	(45.7)	31	(46.3)	108	(47.6)
Not working due to health	11	(9.7)	8	(17.4)	13	(19.4)	32	(14.1)
Cannot find work	30	(26.3)	12	(26.1)	11	(16.4)	53	(23.4)
Not working for other reasons	17	(14.9)	5	(10.9)	12	(17.9)	34	(15.0)
Annual household income <$10,000, n (%)	55	(48.3)	13	(28.3)	38	(56.7)	106	(46.7)

Although all three interventions will be made easily accessible to the target audience and are likely to have an impact, we believe that the IC intervention will most successfully address the needs of low-income smokers. First, the IC program will be personalized and equipped with an intensive, extended counseling and support module provided over the cell phone. Second, the program will combine three intervention components featuring high potential for maintaining support and motivation to quit and prevent relapse. Third, the IC intervention makes use of cell phone technology – an approach that has demonstrated efficacy and feasibility among low-income smokers (i.e., underserved HIV-positive smokers) in previous research conducted by the investigative team [74]. However, we recognize that the IC approach is also expected to be the most expensive; therefore, we will perform an economic evaluation to determine the cost effectiveness of the three treatment approaches.

A major consideration in the design of the study was the potential of the intervention delivery approach to have a significant public health impact. Compared to higher-SES smokers, low-income smokers have limited resources for quitting smoking and are less likely to receive assistance through the health care system [2]. Therefore, it is critically important that these smokers be targeted directly. Thus, we chose to deliver our intervention approaches utilizing a mobile clinic model to provide smoking cessation services to a broad range of underserved populations. If successful and cost-effective, this treatment delivery approach could be easily adopted by other mobile clinics. We believe that the interventions could also be incorporated within a variety of health-related outreach programs targeting low-income individuals. Indeed, dozens of outreach programs exist in the US to address not only cancer prevention but chronic obstructive pulmonary diseases, cardiovascular diseases, diabetes, and other health conditions. For example, according to the Office of Minority Health (personal communication), Texas alone features as many as 52 outreach programs; California has 48 programs. In addition, many health care facilities, universities with medical schools, schools of public health, and other entities invest considerable efforts in reaching the underserved populations. We expect that the interventions and delivery approach would be highly appealing to these entities.

## Abbreviations

US: United States; SES: Socioeconomic status; CDC: Centers for Disease Control and Prevention; NHIS: National Health Interview Survey; ACTION: Adult smoking Cessation Treatment through Innovative Outreach to Neighborhoods; SC: Standard Care; EC: Enhanced Care; IC: Intensive Care; ACASI: Audio Computer- Assisted Self-interview; NRT: Nicotine Replacement Therapy; MI: Motivational Interviewing; FTND: Fagerström Test for Nicotine Dependence; FTQ: Fagerström Tolerance Questionnaire; CES-D: Center of Epidemiologic Studies Depression Scale; PANAS: Positive and Negative Affect Schedule; ISEL: Interpersonal Support Evaluation List; WSWS: Wisconsin Smoking Withdrawal Scale; WISDM: Wisconsin Inventory of Smoking Dependence Motive; MNWS: Minnesota Nicotine Withdrawal Scale; SSS: Subjective Social Status; BRFSS: Behavioral Risk Factor Surveillance System; PSS: Perceived Stress Scale; S-TOFHLA: Short version of the Test of Functional Health Literacy in Adults; SAHLSA: Short Assessment of Health Literacy for Spanish Adults; REALM: Rapid Estimate of Adult Literacy in Medicine; SEM: Structural Equation Modeling; CONSORT: Consolidated Standards of Reporting Trials.

## Competing interests

There are no competing interests.

## Authors' contributions

DV and AP conceptualized and designed the study and were involved in drafting the manuscript. FF conceptualized and prepared the manuscript draft, as well as contributes to study implementation. HD leads study coordination and contributed to study design and drafting the manuscript. SM, JV, and SC contributed to study design and were involved in drafting the manuscript. All authors read and approved the final manuscript.

## Funding

Project ACTION is funded by the National Institutes of Health/National Cancer Institute (R01CA141628) to Alexander V. Prokhorov and Damon J. Vidrine (Multiple Principle Investigators).

## Pre-publication history

The pre-publication history for this paper can be accessed here:

http://www.biomedcentral.com/1471-2458/12/696/prepub
